# 1563. Incidence of Errors in an INSTI-Based Era: A Comparison of Error Rates Between Single Versus Multiple Tablet INSTI-Based Regimens in the Inpatient Setting

**DOI:** 10.1093/ofid/ofad500.1398

**Published:** 2023-11-27

**Authors:** Kayla Blackmon, Denise Kelley, Dusten T T Rose, Brian L Nguyen

**Affiliations:** Ascension Seton, Austin, Texas; Ascension Seton, Austin, Texas; Dell Seton Medical Center at the University of Texas, Austin, Texas; Ascension Seton, Austin, Texas

## Abstract

**Background:**

Errors related to antiretroviral therapy (ART) occur in up to 86% of hospitalized patients living with human immunodeficiency virus (HIV) and may contribute to treatment failure, increased drug resistance, adverse effects, and toxicity. ART can be administered as a single tablet regimen (STR) or multiple tablet regimen (MTR), with limited data on whether the number of tablets affects inpatient error rates. The purpose of this study is to evaluate the incidence of medication errors in adult patients admitted to an Ascension Seton hospital who are maintained on a bictegravir-based STR or a MTR with dolutegravir.

**Methods:**

This is a multicenter, retrospective, observational study conducted in patients ≥18 years diagnosed with HIV and admitted to an Ascension Seton hospital between January 1, 2017 through September 1, 2022. Patients receiving the STR bictegravir-emtricitabine-tenofovir alafenamide fumarate or provided a dolutegravir(DTG)-based MTR (in place of the patient’s home DTG-based STR) while admitted will be included. DTG-based MTRs including lamivudine, or abacavir and lamivudine will be evaluated. The primary outcome is a composite ART-related error rate between groups. Statistical analysis of the primary outcome was evaluated using a chi-squared test with an α level of 0.05 for significance. Secondary outcomes include: each error type overall and between the two groups; time to error correction, defined as time between error occurrence and resolution of identified ART-related errors; and an acquisition cost-analysis of anticipated differences between dispensing DTG-based regimens as MTR versus commercially available STR.

**Results:**

Overall, 726 patient encounters were screened and 514 patient encounters were included (257 encounters in each arm). Patients included in the study were 79.4% male and 48 years of age on average. The primary outcome occurred in 59/257 (23%) encounters in the STR bictegravir group compared to 81/257 (31.5%) encounters in the MTR dolutegravir group (p = 0.029). A total of 68 and 99 errors were identified in the bictegravir and dolutegravir groups, respectively.

**Conclusion:**

Composite error occurrence was significantly higher in the MTR dolutegravir group compared to the STR bictegravir group.

**Disclosures:**

**All Authors**: No reported disclosures

